# Combination of Sodium Selenite and Doxorubicin Prodrug Ac-Phe-Lys-PABC-ADM Affects Gastric Cancer Cell Apoptosis in Xenografted Mice

**DOI:** 10.1155/2019/2486783

**Published:** 2019-08-19

**Authors:** Bo Wu, Jian Ge, Zixiong Zhang, Chuying Huang, Xiaodan Li, Zifu Tan, Xiaojun Fang, Jianhua Sun

**Affiliations:** ^1^Hubei Selenium and Human Health Institute, The Central Hospital of Enshi Tujia and Miao Autonomous Prefecture, Enshi, Hubei 445000, China; ^2^Department of Oncology, The Central Hospital of Enshi Tujia and Miao Autonomous Prefecture, Enshi, Hubei 445000, China; ^3^Department of Gastrointestinal Surgery, The Central Hospital of Enshi Tujia and Miao Autonomous Prefecture, Enshi, Hubei 445000, China

## Abstract

The incidence of gastric cancer is extremely high in China, prompting the development of effective therapeutic strategies. Sodium selenite (SS) affects the proliferation and redifferentiation of gastric cancer cells and the Adriamycin prodrug Ac-Phe-Lys-PABC-ADM (PADM) reduces toxicity in gastric cancer treatment. However, the mechanisms involved therein remain unclear. In this study, nude mice were transplanted with SGC-7901 gastric cancer cells to construct a tumor xenograft model. After administration of SS and PADM, tumor weight and size were reduced. In addition, the levels of alanine aminotransferase, aspartate transaminase, creatinine, and lactate dehydrogenase were decreased, indicating improved hepatic and renal function and inhibited cancer cell metabolism. Furthermore, combined treatment of SS and PADM downregulated the expression of cell cycle-related proteins (cyclin-dependent kinase 4, Ki67, cyclin E, and cyclin D1), elevated that of proapoptosis proteins (Bax, cleaved caspase-3, cleaved caspase-9, and P53), and upregulated that of mitochondrial apoptosis-associated proteins (apoptotic protease activating factor 1 and second mitochondria-derived activator of caspases). In conclusion, combined treatment of SS and PADM effectively promoted apoptosis in gastric cancer xenografts via the mitochondrial apoptosis pathway.

## 1. Introduction

The incidence of gastric cancer is extremely high in China, posing a severe public health problem. The strong heterogeneity of gastric cancer [1, 2] has resulted in a low rate of successful treatment via strategies such as surgery, chemotherapy, radiotherapy, targeted therapy, and immunotherapy. Improving the efficacy of treatment is an issue that needs to be addressed urgently.

Many well-established chemotherapeutic agents have been applied to combat cancer, including doxorubicin (or Adriamycin, ADM), an anthracycline antibiotic. ADM is one of the most important first-line chemotherapeutic drugs with an effective rate of 40–50% in cancer treatment. However, its clinical application is limited because its toxic effects increase with increasing dose [3, 4]. The newly developed doxorubicin/Adriamycin prodrug, Ac-Phe-Lys-PABC-ADM (PADM), is an effective and low-toxicity chemotherapeutic drug that exhibited high ADM targeting. PADM has been shown to be effective in the treatment of gastric cancer with low toxicity [5] and exhibits potential as a chemotherapeutic agent.

Other specific compounds have been shown to exert promising effects against cancer growth. Among them, selenium is a trace element found in selenoproteins, which contain the selenium-based amino acid selenocysteine, and plays an important role in maintaining a healthy human body. Under normal physiological conditions, high selenium content in the serum is positively related to survival rate, while a decrease in serum selenium levels increases the risk of cancer and death [6]. In particular, sodium selenite (SS) is a common inorganic selenium that triggers superoxide anions to induce apoptosis in cancer cells [7, 8], demonstrating its potential anticancer activity.

In this study, SS was administered in combination with PADM to treat nude mice subjected to xenografting of human gastric cancer cells. The tumor conditions and liver function of the experimental mice and the proliferation and apoptosis of gastric cancer cells were examined to explore the combined effect and mechanism of SS and PADM on gastric cancer.

## 2. Materials and Methods

### 2.1. Animals and In Situ Tumor Xenograft

All animal procedures in this study were approved by the Animal Ethics Committee of Wuhan Myhalic Biotechnology Co., Ltd., and performed based on the “Guidelines for Animal Care and Use of the Model Animal Research Institute” (approval number HLK-20181030-01). Six-week-old male BALB/c nude mice were purchased from Huafukang (Beijing, China). SGC-7901 gastric cancer cells (1 × 10^7^ cells/mL) in the logarithmic growth period were digested and resuspended in RPMI-1640 culture medium without fetal bovine serum. The SGC-7901 cell suspension (0.1 mL) was injected into the right dorsal subcutaneous tissues of the mice. After seven days, when the subcutaneous tumor reached a diameter of 0.5–1.0 cm, tumor tissues were removed, cut into 1-mm^3^ blocks, and stored in sterile phosphate-buffered saline at 4°C. Another 24 nude mice were intraperitoneally injected with 1% pentobarbital sodium solution. After anesthesia, the left upper abdomen was conventionally opened through the incision of the rectus abdominis. The entire stomach was exposed and the distal stomach was extracted to form a 3-mm-long wound by rubbing the serosal surface with ophthalmic forceps near the greater curvature of the stomach close to the antrum. The tumor tissues excised previously were fixed to the wound site of the stomach wall with 7-0 sutures. After treatment, the stomach was placed in the abdominal cavity, and the wound was sutured by conventional double-layer incision. When the mice have recovered fully, they were kept in cages and fed a normal diet.

### 2.2. Experimental Protocol

The mice were randomly divided into four groups (n = 6 per group) and treated as follows: control (intraperitoneal injection of saline); SS (3 mg/kg SS by gastric lavage [9]); PADM (intraperitoneal injection of 10 mg/kg PADM [10]); and SS+PADM (3 mg/kg SS by gastric lavage + intraperitoneal injection of 10 mg/kg PADM). The volume of administration was 10 mL/kg for intraperitoneal injection and 200 *μ*L for gastric perfusion. During the entire experimental period, the general health status of the mice was monitored each day. Before drug treatment, the mice were subjected to routine feeding for 7–10 days. Drug administration was performed on days 10, 18, 26, and 34. From the day of establishment of the in situ tumor xenografts, the mice were weighed once every three days, and the end of the experiment was 40 days after the initial administration. After the mice were anesthetized (pentobarbital, 30 mg/kg, i.p.), they were rapidly sacrificed by dislocation and the animal weight and xenograft size were recorded.

### 2.3. Routine Blood Test

Whole-blood platelets were extracted from mice using an automatic blood cell analysis instrument for routine blood test (MEK-8222K, Nihon Kohden, Tomioka, Japan). Whole-blood indices including alanine aminotransferase (ALT), aspartate transaminase (AST), creatinine (Cr), and lactate dehydrogenase (LDH) were analyzed by an automatic biochemical analyzer (Aeroset-2000, Instrument laboratory, Houston, TX, USA).

### 2.4. Immunohistochemistry

Formalin-fixed, paraffin-embedded tissue blocks were placed at −20°C for at least 30 min to increase the hardness before slicing. The tissue blocks were sliced at a thickness of 4–7 *μ*m. Before primary antibody incubation, the sections were heated, dewaxed, hydrated, and subjected to antigen retrieval and elimination of endogenous peroxidase activity. The sections were then incubated overnight at 4°C with primary antibodies against vascular endothelial growth factor (VEGF, rabbit, 1:100, ab52917), CD34 (rabbit, 1:2500, ab81289), Ki67 (rabbit, 1:100, ab15580), E-cadherin (rabbit, 1:30, ab15148), and D2-40 (mouse, 1:40, ab77854). Then, the sections were incubated with horseradish peroxidase (HRP)-conjugated goat anti-rabbit IgG secondary antibody (1:1000, ab6721) or goat anti-mouse IgG (1:500, ab6789). All antibodies were purchased from Abcam (Cambridge, UK). Staining was performed according to the instructions of a commercial kit (Lab Vision, Fremont, CA, USA). The sections were counterstained with Harris' hematoxylin and evaluated by visual assessment of the staining intensity using a microscope at 200×.

### 2.5. Western Blot

Tissues were lysed with radioimmunoprecipitation assay buffer (Thermo Fisher Scientific, Rockford, IL, USA) containing protease inhibitors (Sigma-Aldrich, St. Louis, MO). Proteins were quantified using a bicinchoninic acid protein assay kit (Thermo Fisher Scientific). The primary antibodies were as follows: cyclin-dependent kinase 4 (CDK4, 1:1000, ab108357), Ki67 (1:5000, ab92742), cyclin D1 (1:10000, ab134175), cyclin E (1:1000, ab33911), B-cell lymphoma 2 (Bcl-2, 1:1000, ab32124), Bcl-2-associated X protein (Bax, 1:1000, ab32503), cleaved caspase-3 (1:500, ab2302), cleaved caspase-9 (1:500, ab2324), P53 (1:500, 131442), apoptotic protease activating factor 1 (APAF1, 1:1000, ab2001), second mitochondria-derived activator of caspases (Smac, 1:1000, ab32023), and GAPDH (21118, CST, Boston, USA). After three washes with phosphate-buffered saline/Tween 20, the membranes were incubated with HRP-conjugated secondary goat anti-rabbit IgG (1:2000, ab6721), treated with enhanced chemiluminescence reagent (Millipore, Billerica, MA, USA), and quantified using the Molecular Imager ChemiDoc XRS+ System (Bio-Rad, Hercules, CA, USA).

### 2.6. Statistical Analysis

All statistical analysis was conducted using SPSS 19.0 software (SPSS, Inc., Chicago, IL, USA). Experiments were carried out with three or more replicates. Two-tailed Student's t-tests were used to analyze the results. The data are expressed as mean ± standard deviation (SD). A P value of < 0.05 was considered statistically significant.

## 3. Results

### 3.1. Xenograft Tumor Development Was Inhibited by Combined Treatment of SS and PADM

**Figure 1:**
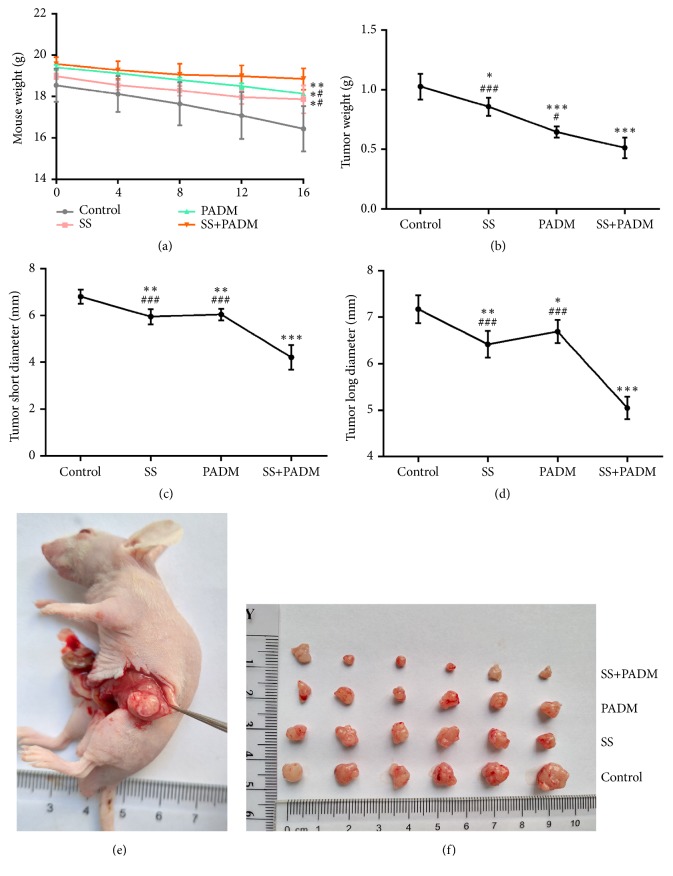
*Xenograft tumor development was inhibited by combined treatment of SS and PADM.* (a) Mouse weight, (b) tumor weight, (c) short diameter, and (d) long diameter. (e, f) Photos of xenografted tumors. All data are presented as the mean ± SD, n = 6. *∗P <* 0.05, *∗∗P <* 0.01, *∗∗∗P <* 0.001* vs.* control; ^#^*P <* 0.05, ^##^*P <* 0.01, ^###^*P <* 0.001* vs.* SS+PADM.

Mice were treated with drugs according to the experimental protocol. The mouse weight, xenograft weight, and long and short diameters of the xenograft were monitored throughout the experimental period. Combined treatment of SS and PADM dramatically increased the weight of the mice (P < 0.05) compared with that of nontreated mice (control), and the combined effect was greater than that of individual administration of SS or PADM (P < 0.05) (Figure 1(a)). The weight, short diameter, and long diameter of the xenograft decreased significantly with combined SS and PADM treatment (P < 0.001) (Figures 1(b)–1(d)), as confirmed by visual observations of the tumors (Figures 1(e) and 1(f)).

### 3.2. Whole-Blood Indices Were Markedly Altered by Combined Treatment of SS and PADM

**Figure 2:**
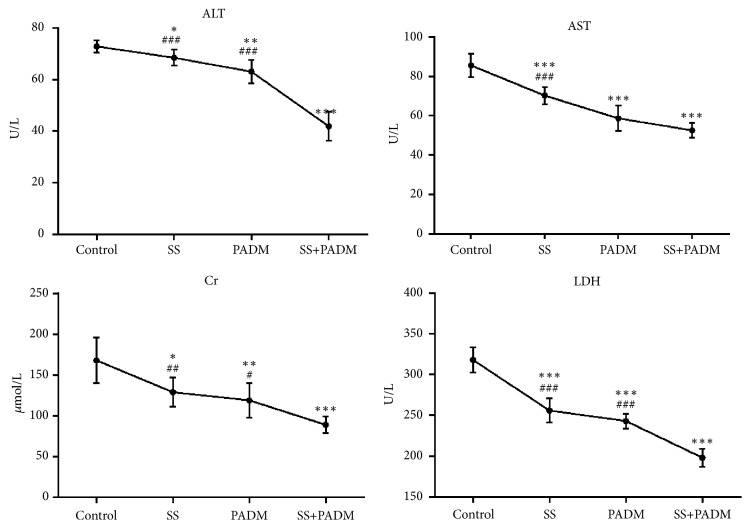
*Whole-blood indices were markedly altered after combined treatment of SS and PADM.* The content of whole-blood ALT, AST, Cr, and LDH. All data are presented as the mean ± SD, n = 6. *∗P <* 0.05, *∗∗P <* 0.01, *∗∗∗P <* 0.001* vs.* control; ^#^*P <* 0.05, ^##^*P <* 0.01, ^###^*P <* 0.001* vs.* SS+PADM.

The levels of whole-blood ALT, AST, Cr, and LDH were detected by an automatic biochemical analyzer. ALT, AST, Cr, and LDH decreased significantly after combined treatment of SS and PADM (P < 0.001) (Figure 2). The combined effect was greater than that of individual administration of SS or PADM.

### 3.3. Immunohistochemistry of Proteins Related to Tumor Development

**Figure 3:**
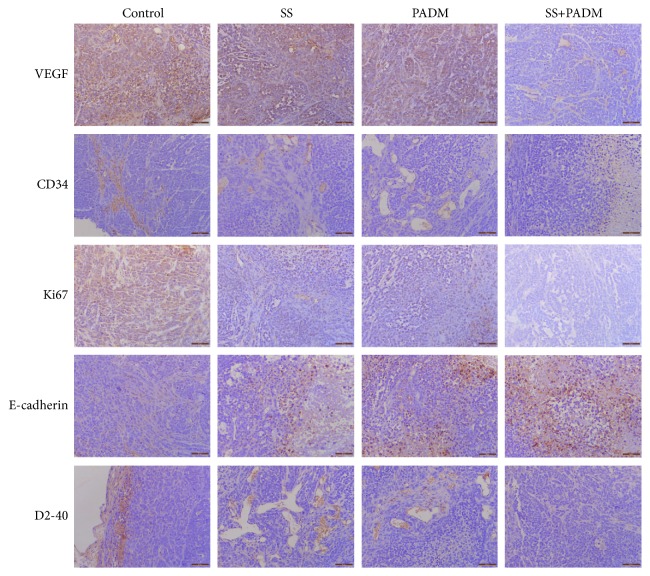
*Immunohistochemistry of proteins related to tumor development.* Proteins related to xenografted tumor development (VEGF, CD34, Ki67, E-cadherin, and D2-40) were detected by immunohistochemistry. Scale bar = 50 *μ*m.

Immunohistochemical staining was performed for VEGF, CD34, Ki67, E-cadherin, and D2-40 to evaluate tumor malignancy. With single SS or PADM treatment, the expression of E-cadherin was increased while that of the other four indicators decreased. The combined effect of SS or PADM was greater than that of individual administration of each compound (Figure 3).

### 3.4. Western Blot of Proteins Associated with Cell Cycle and Apoptosis

**Figure 4:**
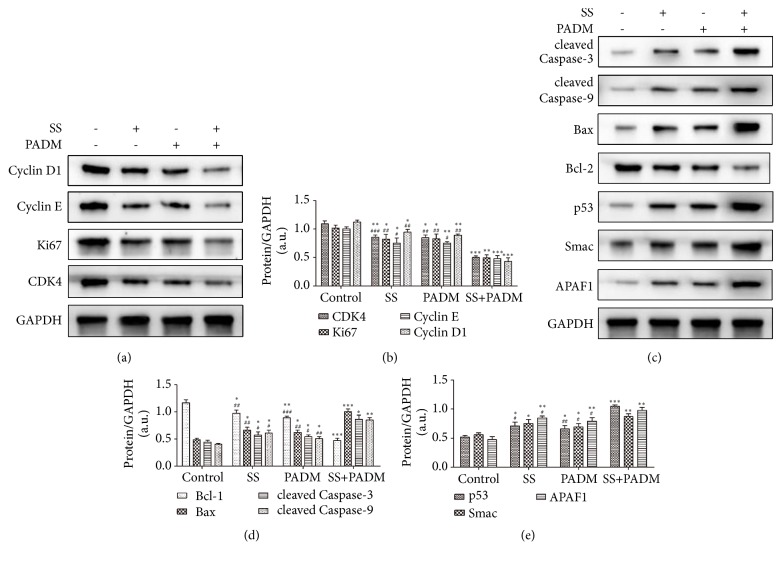
*Western blot of proteins associated with cell cycle and apoptosis.* (a, b) Western blot and quantification of cell cycle-associated proteins (cyclin D1, cyclin E, Ki67, and CDK4). (c–e) Western blot and quantification of apoptosis-related proteins (cleaved caspase-3, cleaved caspase-9, Bax, Bcl-2, and P53) and mitochondrial apoptosis-related proteins (Smac and APAF1). All data are presented as the mean ± SD, n = 3. *∗P <* 0.05, *∗∗P <* 0.01, *∗∗∗P <* 0.001* vs.* control; ^#^*P <* 0.05, ^##^*P <* 0.01, ^###^*P <* 0.001* vs.* SS+PADM.

Proteins related to cell cycle progression (cyclin D1, cyclin E, Ki67, and CDK4) (Figures 4(a) and 4(b)), apoptosis (cleaved caspase-3, cleaved caspase-9, Bax, Bcl-2, and P53) (Figures 4(c) and 4(d)), and mitochondrial apoptosis (Smac and APAF1) (Figures 4(c) and 4(e)) were assessed by western blot. Compared with the nontreated control, combined SS and PADM treatment markedly suppressed all cell cycle-associated proteins, increased the expression of all apoptosis-related proteins aside from Bcl-2, and upregulated both mitochondrial apoptosis-associated proteins.

## 4. Discussion

SS is a readily available and inexpensive drug for cancer treatment and prevention. It has reportedly exerted a variety of effects on tumors, such as lowering the volume of Ehrlich ascites tumors [11], inhibiting the growth of SW480 human colon cancer xenografts [12], and reducing the size and weight of subcutaneously transplanted SGC-7901 tumors in mice [9]. There are many explanations for the anticancer properties of selenium, one of which is the oxidation of thiols in proteins, leading to conformational changes that may weaken the enzymatic activity involved in the metabolism of cancer cells. SS, but not selenate, inhibits protein disulfide exchange reactions, thus preventing the formation of parafibrin, a hydrophobic polymer. Parafibrin can specifically form a protein coating around tumor cells, which is completely resistant to lymphocytic protease-induced degradation such that cancer cells are protected by the body's immune system [13]. Additionally, selenite (Se^+4^) can carry out redox reactions with a protein's sulfhydryl groups expressed on the surface of tumor cells. In this way, selenite can prevent nonenzymatic formation of parafibrin produced by the tumor cells, thus presenting them as “self” and entering the innate cellular immune system [13, 14]. Combined carmustine and selenite treatment significantly inhibited the transmission and proliferation of epidermal growth factor receptor signals and induced apoptosis in androgen-independent prostate cancer cells, suggesting their potential in castration-resistant prostate cancer therapy [15]. These observations indicating that SS reduced the weight of tumor xenografts are consistent with the results of this study.

It has been reported that while SS has synergistic effects with some drugs [16], it exhibited mutual inhibition with certain other drugs [11]. The combined effect of doxorubicin/ADM and other drugs in vivo was impaired in a mouse model of metastatic breast cancer, whereby no evident inhibitory effect on tumor growth was found [17]. ADM-conjugated dendrimers promoted controlled and prolonged exposure of lung-resident cancers to cytotoxic drugs, and PEGylated dendrimers have been suggested as inhalable drug delivery systems to improve anticancer activity [18]. Confocal laser scanning microscopy revealed that compared with control tumors, those exposed to ultrasound exhibited increased uptake of released doxorubicin [19]. The results of this study demonstrated the synergistic activity of SS and PADM, which is not consistent with the results of some studies. This may be explained by the specific interaction between the drugs and their mechanisms of action, which require further investigation.

Despite the promising effects of combined SS and PADM therapy, both components have a certain degree of physiological toxicity, which should not be neglected. The present study showed that both AST and ALT, which are indicators of hepatotoxicity [20], declined after the combined administration of SS and PADM (Figure 2), suggesting that combined treatment may reduce the toxicity of SS and PADM. Meanwhile, impaired renal function that accompanies cancer [21], as evidenced by elevated levels of Cr (Figure 2), was reversed by combined SS and PADM treatment, with a corresponding decline in serum Cr.

Metabolic changes are the most prominent characteristic of cancer cells that involve aerobic glycolysis of lactic acid and higher glucose intake. Cancer cells produce large amounts of energy through aerobic glycolysis, of which LDH is a key regulator that catalyzes the conversion of pyruvic acid into lactic acid [22]. LDH is an important control point in the cell energy release system and its upregulation is common in many malignant tumors. Inhibition of LDH activity showed an antiproliferative effect on cancer cells and reversed their resistance to conventional chemotherapy and radiotherapy [22]. In addition, oxamate-mediated inhibition of LDH induced protective autophagy in gastric cancer cells [23]. The present study suggested that individual administration of SS or PADM reduced the level of LDH (Figure 2), but the effects were accentuated by combined treatment, effectively inhibiting gastric cancer cell proliferation and promoting cell apoptosis.

The suppression of tumor growth by combined SS and PADM treatment was also demonstrated by immunohistochemistry of a variety of factors that participate in tumor progression (Figure 3). VEGF is the strongest and most specific vascular growth factor inducing tumor angiogenesis [24], whereas CD34 has critical functions in intercellular adhesion and the transport and colonization of hematopoietic stem cells [25]. A significant correlation exists between VEGF and CD34, and their coexpression may be an effective indicator of the risk of perioperative hemorrhage in gastric cancer patients [26]. The decreased expression of VEGF and CD34 by combined SS and PADM treatment suggests the inhibition of angiogenesis and perioperative hemorrhage, which reveals positive therapeutic impact. Furthermore, tumor cell proliferation was suppressed by combination treatment, as indicated by the downregulation of Ki67, a proliferating cell-associated nuclear antigen that is directly correlated with gastric cancer progression [27, 28].

In addition, E-cadherin is implicated in selective cell aggregation [29] and was proven to be a tumor suppressor [30]. It is a predictor of overall survival and a marker of metastasis in Asian patients with gastric cancer, and reduced E-cadherin expression is associated with an increased risk of gastric cancer [31]. The expression of E-cadherin was increased by combination treatment of SS and PADM, indicating protection against gastric cancer malignization. Podoplanin (D2-40) specifically labels lymphatics without marking blood vessels to clearly distinguish lymphatic vessels from vessels in tissue [32]. High lymphatic vessel density measured by D2-40 is closely associated with lymph node metastasis in primary gastric tumor specimens and indicates poor clinical outcome. Likewise, the increase in D2-40 immune response in tumors is a putative predictor of aggressive gastric cancer behavior [33]. Combination treatment of SS and PADM effectively attenuated D2-40 expression in tumor xenografts, suggesting that the malignancy was alleviated to a certain extent.

Further observations indicated that individual and combined treatment of SS and PADM decreased the protein expression of cell cycle-associated factors cyclin D1, cyclin E, Ki67, and CDK4. These results suggested that the combination of SS with PADM suppressed the proliferation of gastric cancer cells through regulating cell cycle progression in xenografted tumors. Moreover, the apoptosis-associated proteins cleaved caspase-3, cleaved caspase-9, Bax, Bcl-2, and P53 were examined. The expression of antiapoptotic Bcl-2 was downregulated while those of proapoptotic cleaved caspase-3, cleaved caspase-9, Bax, and P53 were upregulated with combined SS and PADM treatment. Concurrently, the mitochondrial apoptosis-related proteins Smac and APAF1 [34] were also upregulated. These data collectively suggested that combined treatment of SS and PADM strongly promoted cell apoptosis in gastric cancer xenografts, and the involvement of mitochondrial apoptosis therein may be crucial.

## 5. Conclusions

This study demonstrates that the therapeutic potential of SS could be utilized in conjunction with PADM as an alternative to conventional chemotherapeutic drugs such as ADM, and the two compounds showed clear synergistic anticancer effects in an in vivo xenograft model. However, the specific molecular mechanisms that participate in SS-mediated antitumorigenesis, especially those related to mitochondrial apoptosis, need to be investigated in future research in order to take full advantage of the power of combination therapy. Collectively, these findings provide a favorable approach for the treatment and management of gastric cancer and point to combination therapy as a promising direction in anticancer strategies.

## Data Availability

The data used to support the findings of this study are available from the corresponding author upon request.
